# The Association of Physical Activity during Weekdays and Weekend with Body Composition in Young Adults

**DOI:** 10.1155/2016/8236439

**Published:** 2016-04-20

**Authors:** Clemens Drenowatz, Nicole Gribben, Michael D. Wirth, Gregory A. Hand, Robin P. Shook, Stephanie Burgess, Steven N. Blair

**Affiliations:** ^1^Department of Exercise Science, University of South Carolina, Columbia, SC 29208, USA; ^2^Department of Epidemiology and Biostatistics, University of South Carolina, Columbia, SC 29208, USA; ^3^Cancer Prevention and Control Program, University of South Carolina, Columbia, SC 29208, USA; ^4^Connecting Health Innovations, LLC, Columbia, SC 29205, USA; ^5^School of Public Health, West Virginia University, Morgantown, WV 26506, USA; ^6^Department of Kinesiology, Iowa State University, Ames, IA 50011, USA; ^7^College of Nursing, University of South Carolina, Columbia, SC 29208, USA

## Abstract

Physical activity (PA) is a key contributor in long-term weight management but there remains limited research on the association between weekly PA patterns and weight change. The purpose of the present study was to examine the prospective association between weekly PA patterns and weight change in generally healthy young adults. Anthropometric measurements, including dual X-ray absorptiometry, were obtained every 3 months over a period of one year in 338 adults (53% male). At each measurement time, participants wore a multisensor device for a minimum of 10 days to determine total daily energy expenditure and time spent sleeping, sedentary, in light PA (LPA), in moderate PA (MPA), and in vigorous PA (VPA). PA did not differ between weekdays and the weekend at baseline. Twenty-four-hour sleep time, however, was significantly longer during weekends compared to weekdays, which was associated with less time spent sedentary. Weight loss was associated with a significant increase in LPA at the expense of sedentary time during the weekend but not during weekdays. Regression analyses further revealed an inverse association between change in VPA during the weekend and body composition at 12-month follow-up. Taken together, these results suggest that weekend PA plays an important role in long-term weight management.

## 1. Introduction

Obesity is a complex disorder that presents a major threat to future public health due to its association with various chronic diseases, including type 2 diabetes, cardiovascular disease, osteoarthritis, and certain types of cancer, as well as inflammation, a key component involved in the pathophysiology of many chronic diseases [1, 2]. Despite considerable efforts, obesity rates in adults in the United States continue to rise, requiring further research on major contributors to excess weight gain [3]. Emerging evidence suggests that weight gain does not occur consistently. Previous research suggests a more pronounced weight gain during winter months, which has been associated with lower physical activity (PA) levels [4–6]. In addition, weight gain appears to be more pronounced during the weekend compared to weekdays [7], which has been in part attributed to increased energy intake (e.g., greater food and liquid consumption) during the weekend [8–10]. PA has also been shown to vary considerably throughout the week. Evidence on a particular pattern, such as lower PA during the weekend and higher PA during weekdays, however, has been inconsistent [11–14]. Nevertheless, these patterns in weight gain emphasize the importance of behavioral and environmental constraints in weight management [7, 15].

Given the association between PA and body weight [16, 17] it is essential to enhance the understanding of the role of PA patterns in weight management. Despite the availability of objective measurement devices, research on specific PA patterns, particularly regarding weekday and weekend habits, remains limited. Such information, however, appears to be crucial in order to identify specific periods for PA interventions. Intervention strategies may either target times when PA is low or promote an increase in PA, when participants may already be somewhat active [11]. An additional problem of studies examining the association between PA pattern and body weight is the reliance on cross-sectional data [11–13, 18]. Given the bidirectional association between PA and body weight [17], these studies provide only limited insights regarding specific targets for PA intervention strategies. The purpose of this study, therefore, was to examine differences in weekday and weekend PA along with differences in the association between change in PA patterns and change in body weight over a 1-year period. Specifically, it was hypothesized that change in PA levels is higher during the weekend compared to weekdays. Further, it was hypothesized that change in weekend PA has a stronger association with change in body composition compared to change in PA during weekdays.

## 2. Methods

The present study used baseline through 12-month follow-up data from the Energy Balance Study (EBS), a prospective, observational study [19]. The EBS recruited a total of 430 healthy young adults (49% male) between 20 and 35 years of age as there is a particular risk for weight gain at this age range [20]. In order to be included in the study, participants needed to be free of major chronic or acute health conditions without any major changes in their health behaviors in the three months prior to entering the study. Pregnant women, women who were planning on getting pregnant, and those planning to change their use of contraceptive medications in the following 2 years were excluded. The study protocol was approved by the University of South Carolina Institutional Review Board and all participants signed informed consent prior to data collection.

Measurements were conducted every three months by trained and certified research staff. Height (cm) and body weight (kg) were measured with participants in surgical scrubs and bare feet to the nearest 0.1 cm and 0.1 kg, respectively. Body mass index (BMI) was calculated using the average of 3 measures (kg/m^2^). According to current weight management guidelines, a 3% cut-point for change in body weight over 12 months was used to classify participants into weight loss (weight loss > 3%), weight maintenance (weight change ≤ 3%), and weight gain (weight gain > 3%) groups [21].

Total fat mass (FM) and fat free mass (FFM) were assessed via dual X-ray absorptiometry (DXA, Lunar DPX® system, version 3.6; Lunar Radiation Corp., Madison, WI) and percent body fat (% BF) was calculated (FM/body weight). Given the limited accuracy of self-reported dietary intake along with the effect of body weight on reporting accuracy [22, 23], change in FM and FFM during the respective 3-month periods and average total daily energy expenditure (TDEE) was used to calculate energy intake (EI) [24, 25].

TDEE and PA were estimated using the SenseWear Mini Armband (Body Media, Pittsburgh, PA), which participants were asked to wear for a minimum of 10 days every 3 months. Using heat flux, galvanic skin response, skin temperature, near-body ambient temperature, and accelerometry the armband has been shown to provide accurate estimates of energy expenditure, physical activity, and sleep in free-living adults [26–29]. Participants were asked to wear the armband for 24 hours/day and only remove it during periods when it might get wet (e.g., taking a shower or swimming). Compliance was set at 7 days of wear-time, including Saturday and Sunday, and a minimum of 18 hours of wear-time per day. SenseWear's proprietary algorithm (version 7.0 professional, algorithm v2.2) was used to determine TDEE as well as time spent sedentary, excluding sleep (sedentary < 1.5 METs), time spent in light PA (1.5 METs ≤ LPA < 3 METs), time spent in moderate PA (3 METs ≤ MPA < 6 METs), and time spent in vigorous PA (VPA ≥ 6 METs). Data for Saturday and Sunday was averaged to reflect weekend PA and the average from Monday through Friday was used to reflect weekday PA.

During periods of nonwear-time participants reported their activities, which were subsequently matched with the respective energy expenditure based on the Compendium of PA [30] using individual resting metabolic rate (RMR). RMR was measured via indirect calorimetry (True One 2400, Parvo Medics, Sandy, UT) after a 12-hour overnight fast and 24-hour abstention from exercise in a dimly lit room for 45 minutes. RMR was determined as the average of 10 consecutive minutes with the lowest coefficient of variation.

### 2.1. Statistical Analysis

In order to be included in the analysis, valid data needed to be available for at least 3 measurement time points, including baseline and 12-month follow-up. Differences between compliant and noncompliant participants, as well as between weight change groups at baseline, were determined via ANCOVA, adjusting for sex. Differences in TDEE and time spent at different PA intensities between weekdays and the weekend were examined via dependent *t*-tests for the total sample and separately for each weight change group. Linear mixed modelling (LMM) was used to determine change in TDEE, EI, and time spent at different intensities as this allows for an analysis of unbalanced observations over time. Significant changes over the 12-month observation period in TDEE, EI, and time spent at different intensities were examined for the total sample and separately for each weight change group. LMM was further used to determine the association of change in TDEE and PA during weekdays and weekends with subsequent body composition on a continuous level. A subsequent model included sex and change in EI as additional covariates. In order to account for differences in weekday behaviors [11], statistical analyses also were conducted using Saturday and Sunday separately. Statistical analyses were conducted using IBM SPSS Statistics for Windows (version 21.0; IBM Corp., Armonk, NY, USA) with significance set at *p* < 0.05.

## 3. Results

A total of 338 young adults (53.4% male) provided valid data for body composition, TDEE, and PA measures throughout the one-year observation period. On average participants included in the analyses provided PA data for 50.0 ± 3.4 days with an average wear-time of 23.0 ± 1.0 hours/day over the 12-month observation period. Two-thirds of the participants (66.6%) were white with the majority (86.7%) having a college degree. There was no difference in descriptive characteristics at baseline between those included in the analyses and those excluded due to missing follow-up data or noncompliance with the activity measurement. A weight gain of more than 3% (mean gain: 4.4 ± 2.3 kg) was observed in 38.5% of the participants while 13.6% lost more than 3% (mean loss: 4.4 ± 3.0 kg) of their body weight. The prevalence of men was significantly higher in the weight gain group (57.7%), compared to the weight maintenance or weight loss group (43.8% and 43.5%, resp.). There was no difference in ethnicity or education between weight change categories.  Table 1 displays descriptive characteristics for the total sample and separately for weight change categories. Anthropometric characteristics and EI did not differ between weight change categories at baseline after adjusting for sex. There were also no differences in TDEE, in time spent sedentary, and at different PA intensities at baseline between weight change categories after adjusting for sex. TDEE did not differ between weekdays and the weekend even though sedentary time, excluding sleep, was higher on weekdays compared to the weekend at baseline and 12-month follow-up (Table 2). Differences in sedentary time between weekdays and the weekend were due to differences in sleep duration. During waking time participants spent 66.3% and 65.6% in sedentary pursuits on weekdays and the weekend at baseline, respectively. Time spent in light PA, MPA, and VPA did not differ between weekdays and the weekend at baseline and 12-month follow-up. There were, however, significant differences between Saturday and Sunday, with higher activity levels and TDEE on Saturday while Sunday was more sedentary compared to weekdays (Table 3).

Average weight gain for the total sample during the 12-month observation period was 1.2 ± 3.5 kg, which was associated with a significant increase in % BF (1.2 ± 3.5%, *p* < 0.01). Individual weight change ranged from a weight gain of 9.3 kg to a weight loss of 8.3 kg with change in % BF ranging from an increase of 7.0% to a decrease of 8.3%. Despite the significant change in body weight, average TDEE and EI remained constant throughout the observation period. Individual change in TDEE ranged from a decline of 498 kcal/day to an increase of 478 kcal/day. Across the entire sample population, there was a significant reduction in time spent in MPA and VPA (*p* = 0.03) during the 12-month observation period. No difference was observed for 12-month change in TDEE and time spent at different intensities between weekdays and the weekend.

**Figure 1:**
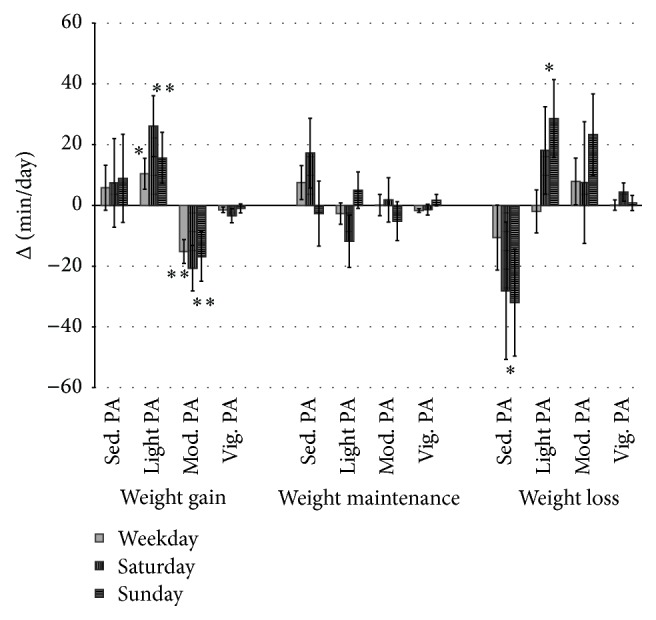
Change in time spent sedentary and different PA intensities from baseline to 12-month follow-up separately for weekdays, Saturday, and Sunday. Values are means with SE. ^*∗*^sig. change within weight change group based on LMM for weekdays or weekend (*p* < 0.05). ^*∗∗*^sig. change within weight change group based on LLM for weekdays or weekend (*p* < 0.01).

Examining weight change categories separately showed a significant decline in TDEE, based on LMM, in the weight loss group during weekdays (ΔTDEE = −106.9 ± 289.2 kcal/day; *p* = 0.02) but not during Saturday or Sunday (ΔTDEE_weekend_ = −12.9 ± 440.7 kcal/day; *p* = 0.84). No change from baseline to follow-up was observed in EI in the weight loss group (ΔEI = −18.8 ± 449.2 kcal/day; *p* = 0.78) or weight maintenance group (ΔEI = −31.9 ± 313.5 kcal/day; *p* = 0.19). There was, however, a significant increase in EI in the weight gain group (ΔEI = 83.3 ± 331.0 kcal/day *p* = 0.01) while TDEE remained constant (ΔTDEE_weekday_ = 32.9 ± 240.8 kcal/day and ΔTDEE_weekend_ = 16.5 ± 369.2 kcal/day; *p* > 0.12). Participants who gained weight, however, reduced time spent in MPA during weekdays, as well as Saturdays and Sundays (*p* ≤ 0.04), while they increased time spent in LPA during weekdays and weekend (*p* ≤ 0.01) (Figure 1). The weight loss group displayed a significant increase in LPA during the weekend (*p* = 0.02) along with a reduction in time spent sedentary (*p* = 0.04). No significant changes were observed during weekdays in the weight loss group. The weight maintenance group did not show a significant change in PA level during weekdays and the weekend.

Examining the association between change in TDEE and subsequent body composition on a continuous level showed no significant association between change in absolute TDEE and body weight at 12-month follow-up. There was, however, a significant direct association between change in TDEE during weekdays and % BF at follow-up (*β* = 0.04, *p* = 0.02) while change in TDEE during the weekend was inversely associated with subsequent % BF (*β* = −0.04, *p* = 0.02). Considering Saturday and Sunday separately in the analysis, only change in TDEE on Sunday remained significant (*β* = −0.04, *p* = 0.03). Results for the regression analyses for time spent sedentary and in different PA intensities and body composition are shown in Table 4. Body weight and % BF at follow-up were inversely associated with change in time spent in MPA during weekdays and the weekend. In addition, change in VPA during the weekend, but not during weekdays, was inversely associated with subsequent body weight and % BF. Further, % BF at follow-up was directly associated with change in time spent sedentary during the weekend. When Saturday and Sunday were considered separately a stronger prospective association between body composition and behavioral choices on Sunday compared to Saturday was observed.  Table 5 further shows that results remained essentially unchanged when EI was included in the regression model.

## 4. Discussion

The health benefits of PA are well recognized [31], but there remains a controversy on the role of exercise for weight loss and weight maintenance. This may in part be due to the variability of PA patterns during the week. Previous research focused predominantly on the cross-sectional association between PA patterns and body weight [11–13, 18], while there are limited data on the prospective association of weekday and weekend PA with weight change. Using 12-month observational data, the present study provides several interesting findings on the interaction between body composition, TDEE, and PA patterns in young adults, who are at particular risk for weight gain [32, 33]. Our results indicate that weekend behavior, particularly on Sundays, has a stronger prospective association with body composition than changes during weekdays. For example, a 30-minute increase in VPA on Sundays was associated with a loss of 0.6 kg or 0.6% body fat over a 1-year period. A 60-minute increase in sedentary time on weekend days, on the other hand, was associated with a 0.5% gain in body fat. In addition, it was shown that weight loss is associated with a reduction in TDEE unless behavioral changes occur. An increase in LPA, at the expense of sedentary time, appears to be sufficient to mitigate the drop in TDEE with a lower body weight. This is of particular importance as low levels of energy expenditure have been associated with a disruption of the regulation of energy balance that facilitate weight regain [34, 35]. The importance of behavioral changes in weight management is further pointed out by a lack of significant change in TDEE with weight gain. Rather, participants who gained weight displayed a significant reduction in MPA while increasing time spent sedentary. A reduction in sedentary time, on the other hand, was associated with beneficial changes in body composition. The fact that these results remained essentially unchanged after controlling for energy intake provides additional support for the importance of MPA and VPA in weight management.

The primary focus, of the study, however, was on examining differences in the association between body composition and PA during weekdays and the weekend. Results highlight the importance of leisure time PA in weight management as associations between PA and body composition were stronger during the weekend compared to weekdays. Particularly Sundays, which are generally the most sedentary days, appear to provide valuable options for an increase in PA and the associated beneficial changes in body composition. The importance of weekend behavior for successful weight loss was also emphasized by results from a weight loss study [10]. The larger influence of weekend PA on body composition potentially reflects the greater flexibility regarding conscious decisions on PA engagement during weekends. PA patterns during the week, on the other hand, are more strongly dictated by occupational demands. Even in the absence of a periodic work rhythm, as experienced by those with a full-time job, people tend to develop specific daily activity patterns in order to adjust to activities of others and opening hours of various businesses [15]. The larger difference in PA levels between normal weight and overweight/obese participants on the weekend compared to weekdays may also be attributed to the greater freedom for behavioral choices during weekends [18]. Nevertheless, it has been argued that total PA remains relatively stable over the entire week [12, 36, 37]. The present study did not show any differences in average PA between weekdays and the weekend. Differences between Saturday and Sunday, however, indicate large interday variability, which may also explain the stronger association between behavioral changes during Sundays and subsequent body weight and body composition. The importance of weekend PA in general may at least partially be attributed to differences in dietary pattern between weekdays and the weekend as weekends have been associated with lower diet quality and higher caloric intake [8–10]. Accordingly, Orsama et al. showed an increase in body weight during the weekend compared to weekdays [7]. Participants, who maintain their body weight, potentially offset this positive energy balance during the weekend with a negative energy balance during weekdays [8]. In addition, results of the present study suggest that participants who lose weight may avoid a positive energy balance during the weekend by increasing LPA while reducing sedentary time. Alterations in LPA also are more reflective of lifestyle choices rather than participation in specific exercise regimen. Such minor changes could have important implications in long-term weight management as a substitution of one hour of sedentary time with activities of light intensity has been suggested to increase energy expenditure in a similar amount as a 15-minute brisk walk [11]. Accordingly, Scheers et al. advocate for an increase in incidental activities in order to reduce sedentary time [11].

Differences in sleep duration between weekdays and weekend days should be considered as well. Previous research indicates that alterations in sleep pattern throughout the week, which is referred to as social jetlag, are associated with increased body weight and adiposity [38, 39]. This may partly be due to lower PA levels in young adults with greater social jetlag (i.e., larger difference in sleep pattern between weekdays and the weekend) [40]. In the present study, participants had significantly longer 24-hour sleep during the weekend, which leads to significant differences in sedentary time between weekdays and the weekend while there was no significant difference in PA. Nevertheless, change in VPA on Sundays was inversely associated with subsequent body weight and body composition, despite the small amount of time spent in VPA. The importance of VPA on weight management has also been emphasized by a cross-sectional study, which showed an increased risk for overweight/obesity with lower VPA while there was no significant association between MPA and weight status [41]. Occupational demands could affect participation in VPA as well. Participants engaging in LPA throughout the day may be too tired at the end of the day to engage in more strenuous activities. The role of fatigue at the end of the day also has been highlighted by the fact that MVPA has been shown to occur more often during morning hours [42, 43]. Engagement in MVPA early in the day, however, could be challenging during weekdays due to conflicts with the work schedule. Consistent with findings from the present study, McCarthy also emphasized the importance of weekend behavior in long-term weight management even though this review focused predominantly on dietary intake [8].

Some limitations of the present study should be considered when evaluating the results. The study population consisted predominantly of white, well-educated young adults, which limits generalizability. Demographic characteristics of the study population also may explain the relatively high PA levels and lower prevalence of overweight/obesity compared to the general US population. Further, Saturday and Sunday were assumed to reflect nonworkdays for the entire sample. Given that the majority of participants were associated with the university, this is a plausible assumption but it may not reflect the actual work pattern of each individual participant. Current work schedules, unfortunately, were not obtained. In addition, the reliance on calculated EI did not allow for a differentiation of dietary intake between weekdays and the weekend. Nevertheless, the inclusion of energy intake in the analysis should be considered a strength of this study as it is a crucial contributor to energy balance and weight management. A major strength of the study is the prospective nature with key measurements taken at multiple time points along with high compliance of objective measures as this provides a more accurate reflection of PA pattern throughout the 12-month observation period, rather than relying on only baseline and 12-month follow-up measurements. Finally, the utilization of objective observational data allows for the examination of lifestyle choices rather than evaluating the response to a specific intervention.

## 5. Conclusions

In summary, the results of the present study emphasize the importance of PA in the regulation of body weight, which is consistent with data from the weight loss registry [44]. Weekend behaviors appear to be of particular importance even though overall PA levels were similar between weekdays and weekend. Given the greater freedom for lifestyle choices during the weekend, weekends have been suggested as a major target for increasing PA [18]. Accordingly, McCarthy suggests increasing awareness of the long-term consequences of short-lived weekend lifestyle choices in order to promote healthy behavioral choices during the weekend [8]. Nevertheless, a holistic approach addressing worksite and leisure time PA will most likely provide the largest benefits regarding a change in PA levels as people who are more active during weekdays have been shown to display higher activity levels during the weekend [13]. More research, however, is warranted to determine the role of specific weekday and weekend behaviors, including dietary intake and sleep patterns, in the regulation of body weight.

## Figures and Tables

**Table 1:** Baseline characteristics for the total sample and separately for weight change groups. Values are mean ± SD.

	Total sample (*N* = 338)	Weight loss (*N* = 46)	Weight maintenance (*N* = 162)	Weight gain (*N* = 130)
Age (yrs)	27.8 ± 3.7	28.1 ± 3.9	28.0 ± 3.7	27.4 ± 3.6
Height (cm)	171.8 ± 9.4	171.2 ± 8.9	170.6 ± 9.4	173.4 ± 9.5
Weight (kg)	74.6 ± 13.8	76.5 ± 12.0	73.3 ± 14.8	75.7 ± 13.0
BMI (kg/m^2^)	25.2 ± 3.8	26.1 ± 3.9	25.1 ± 4.1	25.1 ± 3.5
Fat mass (kg)	21.3 ± 8.7	23.6 ± 8.7	21.5 ± 9.1	20.5 ± 8.0
Fat free mass (kg)	53.9 ± 11.0	53.3 ± 10.2	52.3 ± 11.0	56.1 ± 11.0
% Body fat	28.5 ± 9.0	30.6 ± 9.4	29.1 ± 9.1	27.0 ± 8.5
Calculated EI (kcal/day)	2718.5 ± 503.0	2638.0 ± 500.3	2648.4 ± 502.4	2834.4 ± 486.3

Weight loss: loss in body weight > 3%; weight maintenance: change in body weight ≤ 3%; weight gain: gain in body weight > 3%.

EI: Energy intake.

**Table 2:** Total daily energy expenditure and time spent at different physical activity intensities at baseline and 1-year follow-up. Values are mean ± SD.

	Weight loss (*N* = 46)		Weight maintenance (*N* = 162)		Weight gain (*N* = 130)	
	Weekdays	Weekend	Weekdays	Weekend	Weekdays	Weekend
Baseline						
TDEE (kcal/day)	2777.7 ± 559.7	2727.0 ± 583.1	2665.7 ± 489.4	2655.7 ± 543.4	2804.8 ± 522.9	2810.3 ± 559.3
Sleep (min/day)^1^	390.1 ± 49.4	452.4 ± 68.8	393.3 ± 57.5	443.4 ± 68.6	387.7 ± 54.6	434.1 ± 78.8
Sedentary excl. sleep (min/day)^1^	696.0 ± 95.7	648.1 ± 97.3	696.6 ± 100.0	645.2 ± 123.1	707.3 ± 105.8	660.5 ± 125.3
Light PA (min/day)	220.1 ± 62.5	208.9 ± 67.0	218.6 ± 64.7	223.0 ± 80.7	202.8 ± 60.9	200.4 ± 70.7
MPA (min/day)	124.8 ± 70.4	123.3 ± 90.5	123.2 ± 68.7	121.3 ± 80.0	132.3 ± 79.6	134.7 ± 101.2
VPA (min/day)	8.8 ± 13.5	7.0 ± 10.4	8.0 ± 12.4	7.5 ± 14.6	9.8 ± 13.0	10.5 ± 16.2
1-year follow-up						
TDEE (kcal/day)	2641.3 ± 468.4	2677.6 ± 529.1	2627.2 ± 472.3	2657.3 ± 555.1	2846.3 ± 504.0	2852.6 ± 555.6
Sleep (min/day)^1^	399.7 ± 47.1	444.9 ± 71.1	391.2 ± 58.4	448.4 ± 86.0	386.1 ± 60.7	436.9 ± 88.1
Sedentary excl. sleep (min/day)^1^	684 ± 90.5	616.5 ± 116.4	702.7 ± 104.0	642.7 ± 121.4	709.8 ± 93.5	650.8 ± 129.8
Light PA (min/day)	216.5 ± 57.0	231.9 ± 89.7	215.4 ± 64.9	215.2 ± 68.2	217.9 ± 67.1	224.2 ± 91.2
MPA (min/day)	129.7 ± 61.6	123.7 ± 74.0	123.7 ± 74.0	125.0 ± 87.6	117.1 ± 65.8	119.0 ± 81.5
VPA (min/day)	9.2 ± 12.1	9.9 ± 14.2	6.5 ± 10.8	8.3 ± 15.2	7.9 ± 11.0	8.9 ± 16.5

^1^Significant difference between weekdays and weekend in all weight change categories (*p* < 0.01).

TDEE: total daily energy expenditure; PA: physical activity; MPA: moderate physical activity; VPA: vigorous physical activity.

**Table 3:** Total daily energy expenditure and time spent at different physical activity intensities at baseline during Saturday and Sunday. Values are mean ± SD.

	Weight loss (*N* = 46)		Weight maintenance (*N* = 162)		Weight gain (*N* = 130)	
	Saturday	Sunday	Saturday	Sunday	Saturday	Sunday
TDEE (kcal/day)^1,2,3^	2839.2 ± 698.0	2634.9 ± 540.1	2740.6 ± 599.3	2570.7 ± 556.8	2865.6 ± 645.0	2755.0 ± 568.8
Sleep (min/day)^1,2,3^	421.7 ± 97.7	482.9 ± 90.4	431.8 ± 87.2	455.2 ± 93.4	420.1 ± 104.3	448.4 ± 97.0
Sedentary excl. sleep (min/day)^2^	639.9 ± 134.5	656.2 ± 121.7	623.9 ± 144.4	666.7 ± 135.8	655.3 ± 156.3	666.1 ± 134.9
Light PA (min/day)^1,2,3^	224.9 ± 89.7	192.6 ± 64.0	241.0 ± 112.7	205.3 ± 71.7	210.6 ± 84.3	190.5 ± 79.3
MPA (min/day)^1,2^	142.8 ± 124.1	103.7 ± 70.9	133.2 ± 89.0	109.7 ± 88.9	141.7 ± 114.9	128.0 ± 106.6
VPA (min/day)^1,2^	8.0 ± 15.7	5.7 ± 13.1	9.9 ± 21.0	5.4 ± 13.2	12.8 ± 22.3	8.6 ± 16.8

^1^Significant difference between Saturday and Sunday in weight loss category (*p* < 0.01).

^2^Significant difference between Saturday and Sunday in weight maintenance category (*p* < 0.01).

^3^Significant difference between Saturday and Sunday in weight gain category (*p* < 0.01).

TDEE: total daily energy expenditure; PA: physical activity; MPA: moderate physical activity; VPA: vigorous physical activity.

**Table 4:** The association of 12-month change in time spent at different intensities during weekdays and weekend with body weight and % BF at 12-month follow-up. Values are standardized coefficients (*β*), adjusted for sex and the respective baseline measures of body composition and PA.

	Δsedentary (min/day)^1^		Δlight PA (min/day)^1^		ΔMPA (min/day)^1^		ΔVPA (min/day)^1^	
	Weekday	Weekend	Weekday	Weekend	Weekday	Weekend	Weekday	Weekend
12 M weight (kg)	.015	.022	.022	−.007	−.038^*∗∗*^	−.040^*∗∗*^	−.020	−.035^*∗*^
12 M % body fat	.019	.051^*∗∗*^	.022	−.012	−.067^*∗∗*^	−.072^*∗∗*^	−.031	−.061^*∗∗*^

PA: physical activity; MPA: moderate physical activity; VPA: vigorous physical activity.

Δ: 12-month change based on linear mixed modelling.

^1^Separate regression model for the respective PA intensity due to multicollinearity; ^*∗*^significant at *p* < 0.05,  ^*∗∗*^significant at *p* < 0.01.

**Table 5:** The association of 12-month change in time spent at different intensities during weekdays, Saturdays, and Sundays with body weight and % BF at 12-month follow-up. Values are standardized coefficients (*β*), adjusted for sex, EI, and the respective baseline measures of body composition and PA.

	Δsedentary (min/day)^1^			Δlight PA (min/day)^1^			ΔMPA (min/day)^1^			ΔVPA (min/day)^1^		
	ΔWKD	ΔSAT	ΔSUN	ΔWKD	ΔSAT	ΔSUN	ΔWKD	ΔSAT	ΔSUN	ΔWKD	ΔSAT	ΔSUN
12 M weight (kg)	.028	.004	.036^*∗*^	.017	.012	−.029	−.063^*∗∗*^	−.043^*∗∗*^	−.039^*∗∗*^	−.029	−.029	−.031^*∗*^
12 M % body fat	.025	.032^*∗*^	.045^*∗∗*^	.032	.004	−.029	−.080^*∗∗*^	−.065^*∗∗*^	−.041^*∗*^	−.035	−.031	−.045^*∗∗*^

PA: physical activity; MPA: moderate physical activity; VPA: vigorous physical activity.

WKD: weekdays; SAT: Saturdays; SUN: Sundays.

Δ: 12-month change based on linear mixed modelling.

^1^Separate regression model for the respective PA intensity due to multicollinearity; ^*∗*^significant at *p* < 0.05, ^*∗∗*^significant at *p* < 0.01.
